# Bioconversion of Organic Pollutants in Fish-Canning Wastewater into Volatile Fatty Acids and Polyhydroxyalkanoate

**DOI:** 10.3390/ijerph181910176

**Published:** 2021-09-28

**Authors:** Tania Palmeiro-Sánchez, José Luis Campos, Anuska Mosquera-Corral

**Affiliations:** 1 Microbiology, Ryan Institute, School of Natural Sciences, National University of Ireland Galway, H91 TK33 Galway, Ireland; 2 Faculty of Engineering and Science, Universidad Adolfo Ibáñez, Avda. Padre Hurtado 750, Viña del Mar 2520000, Chile; jluis.campos@uai.cl; 3 CRETUS, Department of Chemical Engineering, University of Santiago de Compostela, 15782 Santiago de Compostela, Spain; anuska.mosquera@usc.es

**Keywords:** acidogenic fermentation, fish-canning wastewater, mixed culture, polyhydroxyalkanoates (PHAs), saline conditions, volatile fatty acids (VFAs)

## Abstract

The wastewater from the cookers of a tuna-canning plant was used as feedstock for the process. It was acidified in a continuous stirred tank reactor (CSTR) of 1.5 L to produce a mixture of volatile fatty acids (VFAs). The effluent contained 28.3 ± 8.7 g COD_S_/L and 25.0 ± 4.6 g COD_VFA_/L, 4.4 ± 1.6 g NH_4_^+^/L, and 10.9 ± 4.0 g Na^+^/L, which corresponds to about 28 g NaCl/L approximately. This was used to feed a PHA production system. The enriched MMC presented a capacity to accumulate PHAs from the fermented tuna wastewater. The maximum PHA content of the biomass in the fed-batch (8.35 wt% PHA) seemed very low, possibly due to the variable salinity (from 2.2 up to 12.3 g NaCl/L) and the presence of ammonium (which promoted the biomass growth). The batch assay showed a PHA accumulation of 5.70 wt% PHA, but this is a much better result if the productivity of the reactor is taken into account. The fed-batch reactor had a productivity of 10.3 mg PHA/(L h), while the batch value was about five times higher (55.4 mg PHA/(L h)). At the sight of the results, it can be seen that the acidification of fish-canning wastewater is possible even at high saline concentrations (27.7 g NaCl/L). On the other hand, the enrichment and accumulation results show us promising news and which direction has to be followed: PHAs can be obtained from challenging substrates, and the feeding mode during the accumulation stage has an important role to play when it comes to inhibition.

## 1. Introduction

Fish-canning industries are essential for the local economy in Galicia (Spain), and they also present a significant role in the Spanish market. Just the Galician industries account for the 85% of the economic value of the whole national production of canned fish products and to 87% of the total volume. Even at European levels, the fish-canning companies and other marine industries are gaining importance in their manifest significance as key sectors for the economic growth of the European Union [1], representing about 5.4 million jobs [2].

Beyond the employment generation and the economic growth related to fish-canning and marine industries, this sector produces great amounts of wastes that are not negligible and need further treatment. These effluents are mainly characterized by abundant flows with high organic loads in addition to high saline concentrations [3]. Physico-chemical processes, such as coagulation, flocculation, filtration, etc., usually accomplish the treatment of these saline wastewaters although the operational costs of these processes are elevated [4,5]. In other cases, biological treatments are applied and chosen depending on the desired objective. In this case, these saline effluents can be treated by aerobic or anaerobic biological systems [6,7,8,9,10,11]. It is very challenging to manage fish-canning effluents, as they are seasonal with high fluctuation of organic matter. In addition, there are several streams within a fish-canning factory, with different flows, temperatures, and compositions depending on the part of the process, such as boiling or cleaning [3]. The salinity of this effluents is variable (0.2–3.3 g SO_4_^2−^/L and 1.6–41.5 g Cl^−^/L) and so it is the organic matter content (2.9–120.9 g COD/L) [12]. There are several biological ways to treat fish-canning wastewaters [13]. These effluents are a source of organic matter that can be used for the production of valuable compounds such as biopolymers, fulfilling the requirements of the European Circular Economy [14]. For instance, agri-food waste streams have been reported as suitable substrates for PHA production [15]. In this sense, fish-canning wastewaters seem to be also suitable for PHA production regarding their organic matter content.

Several different residues have been already tested for PHA production in mixed microbial cultures: olive oil mill effluents [16], sugar cane molasses [17,18,19,20], paper mill wastewater [21,22], waste activated sludge [23,24], tomato cannery [25], cheese whey [20], candy-bar industrial effluents [26], etc. Very promising results have been achieved even at pilot scale, reaching accumulation values higher than 70 wt.% PHA when using sugary substrates [26]. However, unlike the fish-canning streams, these effluents are characterised by the presence of carbohydrates—and lipids in the lesser cases—and the absence of significant amounts of proteins or sodium chloride, which are a typical characteristic of fish-canning wastewater. On the one hand, salinity is known to affect the PHA accumulation capacity [27] and the properties of the obtained biopolymer [28] in mixed microbial cultures. On the other hand, relevant concentrations of proteins lead to the presence of considerable amounts of ammonium in the medium of reaction. The excess of ammonium might be detrimental for some bacterial activities. For instance, it is well known that it can lead to inhibition of the anaerobic process. Regarding the PHA production, an excess of ammonium is usually associated with lower accumulation percentages because it affects both the enrichment and accumulation stages. During the enrichment, optimal values for the C/N ratios are within the range of 8–10. For the accumulation batches, ammonium promotes growth, which competes with accumulation and gives lower wt% PHA values if compared with the accumulation under ammonium starvation conditions [29]. Moralejo et al. (2013) found that the ammonium was not consumed in the first hours of the accumulation because storage was promoted over growth [30]. Johnson et al. (2010) and Moralejo et al. (2013) observed opposite behaviours when dealing with an excess of ammonium in the accumulation assays, which indicates that further research is needed in this way.

The aim of the present study is to investigate the feasibility of producing PHAs with fish-canning wastewater. For this purpose, the following steps had to be addressed: (1) characterization of the acidification operation in terms of efficiency and VFA composition; (2) if it is feasible to select a microbial community able to accumulate PHAs using the acidified stream as substrate; (3) evaluate the accumulation performance under high ammonium and saline concentrations. The principal aim of the present study is to investigate the feasibility of PHA production using acidified fish-canning wastewater, containing relevant concentrations of volatile fatty acids, ammonium, and sodium chloride. To the knowledge of the authors, no previous research has been done regarding the PHA production with high saline and high NH_4_^+^ content.

## 2. Materials and Methods

### 2.1. Fish-Canning Wastewater Characterization

Fish-canning wastewater (FCW) was taken from the boilers of the fish-canning factory of Conservas Selectas de Galicia S.L. (O Grove, Spain), right after cooking the tuna, at a temperature close to the water boiling point.

The FCW was characterized in terms of complex organic matter (fats, proteins, and carbohydrates), VFAs, chemical oxygen demand (COD), biochemical oxygen demand (BOD5), total suspended solids (TSS), volatile suspended solids (VSS), and ions (sodium—Na^+^, potassium—K^+^, magnesium—Mg_2_^+^, calcium—Ca_2_^+^, sulphate—SO_4_^2−^, phosphate—PO_4_^3−^, nitrite—NO_2_^−^, nitrate—NO_3_^−^).

### 2.2. Experimental Set-Up

**Figure 1:**
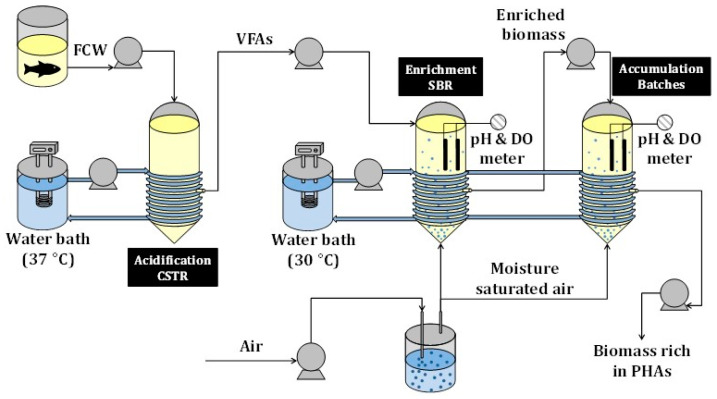
Experimental set-up for the valorisation of FCW into PHAs.

The experimental set-up is shown in Figure 1. It is made up of three stages: (1) acidification of the FCW in a continuous stirred tank reactor (CSTR); (2) enrichment of the accumulating mixed microbial community in a sequential batch reactor (SBR); (3) maximisation of the PHA accumulation in batch and fed-batch tests.

#### 2.2.1. Acidification

The acidification of the FCW was performed in a CSTR with a working volume of 1.5 L. It was a jacketed anaerobic fermentor (Álamo, Spain) set at 35 °C using a water thermostatic bath (Techne Inc., Burlington, NJ, USA) and the mixing was maintained constant at 150 rpm by means of a mechanical stirrer (IKA, Staufen, Germany). The reactor was inoculated with sludge from an anaerobic reactor treating dairy wastewater with the operational conditions described elsewhere [31]. The CSTR was operated for 203 days at a hydraulic (HRT) and solids retention time (SRT) of 2 days. The organic loading rate (OLR) was maintained at 12.3 ± 3.5 g COD/(L · d). During the operation, the pH was measured but not controlled. The other operational parameters (NH_4_^+^, VFAs, COD_T_, COD_S_, TSS, VSS, and ions) were also measured.

#### 2.2.2. Enrichment

The enrichment consists in the selection of accumulating micro-organisms by applying a selective pressure (the limitation of the availability of the carbon source). For this purpose, a 1.8 L SBR (Álamo, Spain) was inoculated with activated sludge (0.68 ± 0.16 g_TSS/L, 0.54 ± 0.12 g_VSS/L) collected from a nearby wastewater treatment plant (Calo, A Coruña, Spain). The reactor was operated for 384 days under non-sterile conditions following a feast–famine sequence. To promote this limitation of the carbon source, the SBR cycles were distributed in a feeding phase (15 min), a reaction phase (675 min), a withdrawal phase (15 min), and an idle phase (15 min). The reactor was fully aerated, and no settling step was included in the SBR cycles with the purpose of having a HRT equal to the SRT, which had a value of 1 day. The temperature was maintained at 30 °C by means of a thermostatic bath (Techne Inc., USA). The dissolved oxygen concentration (DO) was measured onsite during the whole operational period. The pH, NH_4_^+^, VFAs, TS, VS, and ions were also measured during the 384 days of operation and also during the characterization of the enrichment cycles.

#### 2.2.3. Accumulation

The accumulation tests consist in the maximization of the biopolymer inside the cells to determine the maximum storage capacity of the selected microbial community. For this purpose, two different types of accumulation assays were performed: (1) one operated in fed-batch mode and (2) another operated in batch mode. Further details are provided below:

1. Fed-batch assay: The substrate was added in pulses. The complete consumption of the VFAs present in the feeding was noticed by the increase of the DO concentration, which was monitored and measured on-line. Each time the DO concentration rose up, a new pulse of feeding was added. The volume of each pulse was of 40 mL, which corresponded to 28.3 Cmmol VFAs per pulse.
2. Batch assay: A volume of 520 mL of substrate was added just once, which means a solely pulse of approximately 250 Cmmol VFA.

These two different strategies (batch and fed-batch) were compared. Data collected from all accumulation experiments were used to calculate the corresponding stoichiometric and kinetic parameters of each assay. The impact of the presence of NaCl in the media was also closely followed as its concentration was constant in the batch assay but it continuously increased in the fed-batch.

The inoculum used for these tests was freshly harvested from the enrichment reactor at the end of one of the SBR cycles. The accumulation assays were performed under the same environmental conditions of the enrichment (30 °C, fully aerated, stirred). The samples in the fed-batch assay were taken before adding a new feeding pulse. In the case of the batch assay, samples were taken every hour. During the night no samples were taken. The pH, DO, NH_4_^+^, VFAs, TS, VS, and ions were measured during the accumulation experiments.

### 2.3. Analytical Methods

The DO was measured and acquired with an optical luminescent probe on site (Hach, Loveland, CO, USA). The pH was measured with a pH-meter with a glass electrode (Crison, Alella, Spain). The partial (PA) and total alkalinity (TA) were determined at pH 5.75 and 4.3, respectively, by titration with H_2_SO_4_ 0.05 N to calculate the Ripley ratio [32]. Analysis of TS, VS, TSS, and VSS were calculated according to the gravimetric method of the standard methods [33]. The COD was determined by the oxidation of the organic matter with K_2_Cr_2_O_7_ 0.25 N together with a catalyst (H_2_SO_4_ together with Ag_2_SO_4_) and titrated with Fe(NH_4_)_2_(SO_4_)_2_.6H_2_O 0.05 N according to the standard methods [33]. NH_4_^+^ was measured by a spectrophotometric method using the salicylate–hypochlorite method [34]. Ions (Na^+^, K^+^, Mg^2+^, Ca^2+^, SO_4_^2−^, PO_4_^3−^, NO_2_^−^, NO_3_^−^) were analysed by ion chromatography (Metrohm, Switzerland). VFA (acetic—HAc, propionic—HPr, butyric—HBu, and valeric—HVa) concentrations were analysed by gas chromatography (GC) (Hewlett Packard, Palo Alto, CA, USA) following the standard methods [33]. For the characterization and determination of the accumulated PHAs, biomass samples were analysed by GC (Hewlett Packard, USA) following an esterification method [35]. The samples were taken directly from the corresponding reactors and formaldehyde (37 v%) was added to stop any biological activity. Afterwards, they were frozen before placing them in a freeze-drier. Each lyophilised sample was digested at 100 °C for 2.5 h using a mixture of C_3_H_8_O:HCl 4:1 and 1,2-dichloroethane, using benzoic acid as the standard. In order to extract the esters, water was added and separated from the organic phase, which was analysed in the GC.

### 2.4. Calculations

The intermediate alkalinity (IA) is the difference between the TA and the PA, and it corresponds to the VFA alkalinity [32]. The ratio between the IA and PA is known as the Ripley ratio and calculated as follows (Equation (1)):

Ripley ratio = (TA − PA)/PA = IA/PA(1)

The acidification efficiency (COD%) was calculated as the percentage of the ratio between the COD in the influent (COD_IN_) and the COD due to VFAs in the effluent (COD_VFA_) (Equation (2)). With the aim to convert the VFA concentrations into their COD equivalent, the following values were used: 1.07 mg COD/L for 1 mg HAc/L; 1.51 mg COD/L for 1 mg HPr/L; 1.82 mg COD/L for 1 mg HBu/L; 2.04 mg COD/L for 1 mg HVa/L [36].

Acidification efficiency (COD%) = (COD_VFA_ (g/L))/(COD_IN_ (g/L))·100 (2)

The amount of biomass produced was estimated as a function of the consumed ammonium. Assuming a biomass composition of CH_1.8_O_0.5_N_0.2_ [37], it was supposed that every Nmol of NH_4_^+^ consumed led to 5 Cmol of biomass.

The PHAs were characterized in the GC by the content of the monomers hydroxybutyrate (HB) and hydroxyvalerate (HV). The amount accumulated in the biomass samples was calculated as a percentage in dry weight (wt%) following Equation (3).

PHA (wt%) = (PHA (g))/(TS (g))·100(3)

The productivity was calculated as a function of the amount of PHA produced over time depending on the volume of the reactor following Equation (4).

Productivity (g PHA/(L h)) = (average total amount of PHA produced in the reactor per unit of time (g))/(Reactor volume (L) · time (h))(4)

The specific PHA production (Cmol PHA/(Cmol X h)) and VFA uptake ((Cmol VFA/(Cmol X h)) rates were determined by linear regression. The data obtained from the enrichment cycles and the accumulation tests were plotted representing the amount of PHA produced and the VFAs consumed against time and taking into account the average biomass along the experiment. These data were also used for the determination of PHB and PHV yields (Cmol PHx/Cmol VFA) by dividing the specific PHB and PHV production rates by the specific VFA uptake rate.

## 3. Results and Discussion

### 3.1. Acidification

The wastewater collected from the tuna cookers was fermented anaerobically to produce VFAs. The fatty acids served as substrate for PHA production at a later stage (Figure 1). The waste stream from the tuna cookers had a high content in organic matter and salinity (Table 1). In addition, the protein concentration in the influent was not negligible and large amounts of NH_4_^+^ were expected as a result of their anaerobic degradation. Considering these complex circumstances, a detailed performance of the anaerobic acidification reactor was followed during the 203 days of operation. A stirred (150 rpm) and jacketed (35 °C) CSTR operated for 203 days at an HRT of 2 days acidify the waste stream from the tuna cookers of a fish-canning industry (Conservas Selecta de Galicia S.L., Galicia, Spain).

**Figure 2:**
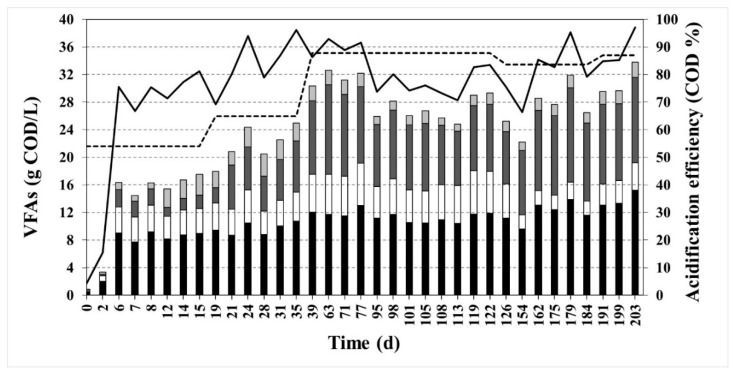
VFA production and distribution of acids in the acidification CSTR along the 203 days of operation. VFA concentration (g COD/L) composition in bars (HAc in black; HPr in white; HBu in dark grey; HVa in light grey); feeding concentration (g COD/L) (discontinuous line); the efficiency of the acidification (COD%) (continuous line).

The pH, the alkalinity, the concentrations of ions, COD, VFAs, NH_4_^+^, TSS, and VSS were measured on a regular basis as indicators of the performance of the fermenter. All the values can be found in Table 2. The pH of the reactor was measured but not controlled and it remained stable during the whole operation at a value of 7.5 ± 0.1. The concentration of ammonium was 4.4 ± 1.6 g NH_4_^+^/L, and the sodium concentration was 10.9 ± 4.0 g Na^+^/L, which corresponds to 27.7 g NaCl/L approximately. TA and PA showed values of 11.5 ± 0.9 and 3.6 ± 0.4 g CaCO_3_/L, respectively, while the Ripley ratio had an average value of 2.3 ± 0.3, which is an indicator that no methane was being produced [32]. After day six, the acidification efficiency reached a value above 70 COD% and the steady state showed an overall efficiency of 81.1 ± 7.0 COD% (Figure 2).

**Figure 3:**
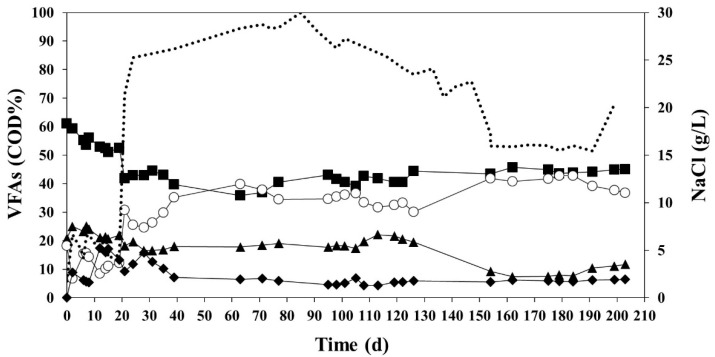
VFA composition (COD%) for HAc (■), HPr (▲), HBu (○), and HVa (♦), together with the concentration of NaCl (dotted line) during the 203 days of operation.

However, the VFA composition (expressed in terms of COD%, Figure 2) reached its steady state later, after 39 days of operation. A closer look to the VFA composition can be seen in Figure 3. Acetic acid was the main product before day 39, but afterwards butyric was also produced in the same amount in terms of COD. It can be observed in Figure 3 that high salinity triggers butyric acid formation as other authors previously observed too [38]. All effluents were mixed in a drum and stored in a cold camera at 4 °C with the objective of being used later for PHA production. The effluent was centrifuged at 16,000 rpm for 15 min at 10 °C before storage to remove most of the bacteria and avoid degradation at low temperatures.

### 3.2. Enrichment of a MMC Using the Effluent of the Acidification Reactor Diluted

The SBR operated for 384 days under the operational conditions shown on Table 3. The enrichment reactor was monitored so as to observe the evolution of the performance of the selected microbial community. The average TS and VS concentrations inside the reactor were 1.34 ± 0.47 g/L and 1.21 ± 0.43 g/L, respectively. The 90.3 ± 5.2% of the TS were VS. The average values of other measured parameters were 256.4 ± 20.3 mg NH_4_^+^/L, 1.20 ± 0.32 g NaCl/L at the end of the famine phase. The reactor showed a feast phase of 155 ± 24 min over the total length of the cycle of 720 min. This means a feast of about 21% of the total time of the cycle. The enrichment showed a stable performance despite the fact that ammonium was present in relatively high concentration in comparison with other conventional enrichment configurations [27,30,39].

**Figure 4:**
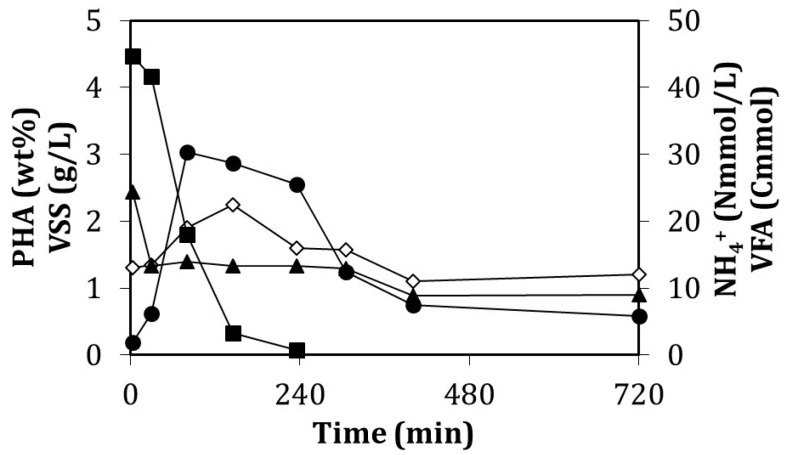
Evolution of the measured compounds during a representative enrichment cycle performed on day 261 of operation. Amount of consumed VFAs (Cmmol) (■); NH_4_^+^ concentration (Nmmol/L) (▲); VSS concentration (g/L) (◊); percentage of PHA accumulated (wt%) (●).

Several enrichment cycles were monitored, which provided data similar to that shown in Figure 4, considered as a cycle representative of the reactor performance. Estimated yields for biomass and CO_2_ were of 0.376 ± 0.065 Cmol X/Cmol VFA and 0.533 ± 0.104 Cmol CO_2_/Cmol VFA, respectively. The yields for PHA production were of 0.099 ± 0.052 Cmol HB/Cmol VFA and 0.016 ± 0.011 Cmol HV/Cmol VFA for each one of the monomers. The estimated average error of the balances was 2.5%.

The specific substrate consumption rate was of 0.386 ± 0.036 Cmol VFA/(Cmol X h) and the specific biopolymer production rate was 0.045 ± 0.019 Cmol PHA/(Cmol X h). The specific growth rate was 0.144 ± 0.023 Cmol/(Cmol X h) and the specific CO_2_ production rate was 0.205 ± 0.035 Cmol CO_2_/(Cmol X h). Nevertheless, Duque et al. (2014) obtained better results when using fermented molasses and fermented cheese whey as feedstock, with a value of 0.25 Cmol PHA/(Cmol X h) for fermented molasses and values of 0.27 Cmol PHA/(Cmol X h) for fermented cheese whey. Duque et al. (2014) explained their good results based on the application of eight cycles per SRT since the selective pressure is increased when lower number of cycles per SRT are performed [40]. Nonetheless, Jiang et al. (2011) used a SRT equal to the HRT of 1 day, the same used in the present work, while Duque et al. (2014) applied an HRT of 1 day and a SRT of 4 days. In this way, it seems that the HRT also have an influence in the selection of the MMC since in the present study, two cycles were conducted per SRT and the PHA accumulation values during the feast phase were low in comparison with the ones obtained by Duque et al. (2014).

### 3.3. Accumulation Performance of a MMC Using the Effluent of the Acidification Reactor without Any Dilution

The accumulation reactor was inoculated with biomass withdrawn from the enrichment reactor at the end of the SBR cycle. The accumulation experiments were performed using as a substrate the effluent of the acidification reactor. These assays had high concentrations of ammonium and sodium chloride. Due to the fact of the presence of a nutrients source, a competition between growth and accumulation for the use of the carbon source was expected.

- Fed-batch accumulation

**Figure 5:**
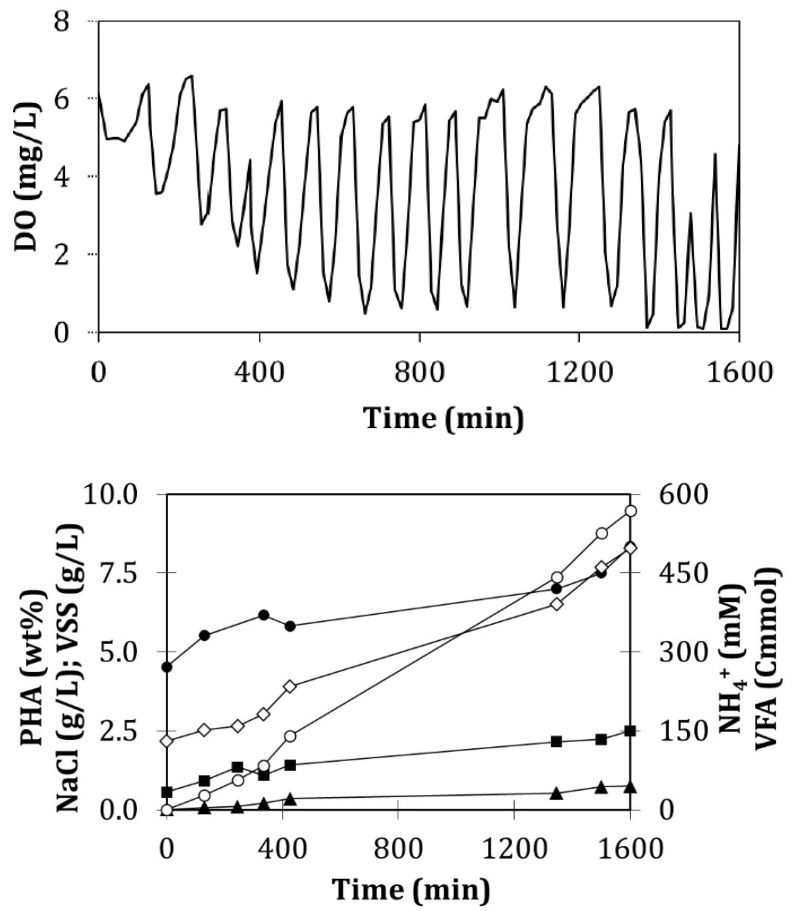
The upper figure is the profile of the dissolved oxygen concentration throughout a fed-batch accumulation assay performed on day 211. The lower figure is the evolution of the concentrations of the measured parameters for the same fed-batch accumulation assay. Cumulative amount of consumed VFAs (Cmmol) (○); NH_4_^+^ concentration (Nmmol/L) (▲); VSS concentration (g/L) (■); NaCl concentration (g/L) (◊); percentage of the stored PHA (wt%) (●).

The growth of active biomass (X) was relevant (Figure 5) as nitrogen was added with each pulse with a specific production rate of 0.07 Cmol X/(Cmol X h). The concentration of NH_4_^+^ in the assay increased from 0.51 to 45.1 mM. The maximum obtained biopolymer content was 8.35 wt% PHA after 27 h of reaction. In productivity terms, this means a value of 10.3 mg PHA/(L h) (Table 4). Due to the observed growth values and the low values of accumulation percentages, it can be assumed that the competition between biomass growth and PHA accumulation occurred. Typically, accumulation assays have no nitrogen addition to avoid biomass growth, which hinders the PHA accumulation. In the present study, the VSS augmented from 0.56 to 2.50 g VSS/L (Figure 5) during the accumulation experiment. This difference between final and initial VSS concentration is attributed to PHA accumulation and biomass growth. However, in the present case, the accumulation percentage increased only from 4.54 to 8.35 wt% of PHA due to the biomass growth. Moreover, kinetic and stoichiometric parameters (Table 5) support this finding since PHA specific production rates (0.005 Cmol HB/(Cmol X h) and 0.001 Cmol HV/(Cmol X h)) are very low in comparison with growth rates (0.068 Cmol X/(Cmol X h)).

Accumulation yields were much higher for growth (0.366 Cmol X/Cmol VFA) than for the PHA accumulation (0.029 Cmol HB/Cmol VFA and 0.005 Cmol HV/Cmol VFA). Not only the presence of ammonia but also the presence of sodium chloride is known to affect the microbial accumulating response of the system. Values of IC50 of approximately 5 g NaCl/L were reported for PHA accumulation in similar enriched MMCs [27]. In the present case, the salt concentration ranged from 2.2 to 8.3 g NaCl/L, indicating that a possible inhibition could take place, influencing the PHA storage.

The PHA accumulation rate was only 0.006 Cmol PHA/(Cmol X h) (Table 5). It can also be observed that HB accumulation was promoted over HV accumulation. This behaviour might be explained by the fact that propionate has higher energy potential production in comparison with acetate when cell maintenance comes along during the inhibiting processes, as it was observed in previous studies [27]. This statement is based on the assumption that butyrate and valerate are not expected to contribute decisively to biomass maintenance [41].

- Batch accumulation

**Figure 6:**
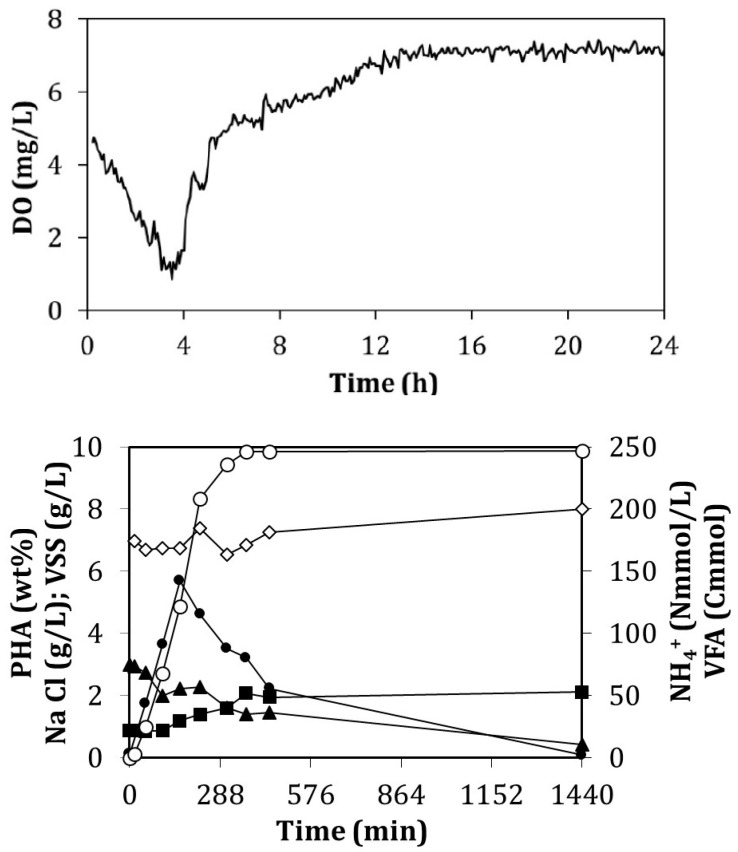
The upper figure is the profile of the dissolved oxygen concentration throughout the batch accumulation assay performed on day 260. The lower figure shows the evolution of the concentrations of the measured parameters for the same batch accumulation assay. Consumed VFAs (Cmmol) (○); NH_4_^+^-N concentration (Nmmol/L) (▲); VSS concentration (g/L) (■); NaCl concentration (g/L) (◊); percentage of the stored PHA (wt%) (●).

The substrate was fed by adding just one single pulse of substrate and it was completely consumed after 6 h. The evolution of the DO concentration was monitored and measured on-line (Figure 6).

The batch assay lasted 24 h although the maximum value of 5.7 wt% PHA was achieved after 2.7 h of the experiment. At this moment, 122 Cmmol VFA had been consumed (Figure 6). After this point, the PHA inside the cells started to diminish (Figure 6) even though half of the added VFAs were still present in the media amounting to approximately 125 Cmmol VFA. Table 4 gives information about the values of several of these parameters. It is important to remark that the growth of active biomass (X) was relevant again at a specific rate of 0.121 Cmol X/(Cmol X h). The concentration of ammonium in the assay decreased from 75 to 56 Nmmol/L in the accumulation period and it also varied from 75 to 10 Nmmol/L during the whole cycle. In productivity terms, the accumulation value of 5.7 wt% is related to a value of 55.4 mg PHA/(L h). The composition of the obtained biopolymer was of 15.9 g/g in terms of HB:HV ratio (Table 4), which was very similar to the value at the beginning of the assay (HB:HV of 15.3 g/g). In this case, both biopolymers were produced in a similar extent. This fact could be due to the constant concentration of 6.81 g NaCl/L throughout the cycle as the micro-organisms were affected more or less to the same extent. After the point of maximum accumulation, both monomers were consumed for maintenance. After 6 h from the beginning of the experiment, the HB:HV ratio was approximately 26 g/g, while after 24 h this value increased tremendously as HV was fully depleted (Table 4).

From the performed accumulation assays, a PHA accumulation of 8.35 wt% PHA was obtained in fed-batch and 5.70 wt% PHA in the batch. The productivity values for each assay were 55.4 mg PHA/(L h) for the batch assay and 10.31 mg PHA/(L h) for the fed-batch one. Both results might appear contradictory, but the excess of ammonium is the responsible of these results. The biomass growth was favoured in the fed-batch assay when the pulse-wise feeding strategy was applied, with a biomass yield of 0.366 Cmol X/Cmol VFA in comparison with the value of 0.226 Cmol X/Cmol VFA obtained when one single pulse was applied. Despite the growth rate being higher in the batch assay (Table 5), it can be affirmed that accumulation is promoted in the batch in comparison with the fed-batch. In fact, the accumulation rate in the one single pulse test was nearly ten times higher and the accumulation yield was doubled. Another important fact is the PHB and PHV composition. Despite the fact that the substrate was the same in both assays, the composition was very different. This might be attributed to both different saline and ammonium concentrations, which affected the microbial activity and hence, the biopolymer composition.

However, it cannot be denied that low kinetic and stoichiometric values were obtained for both assays. The saline inhibition seems the strongest reason for this behaviour but the high concentrations of NH_4_^+^ cannot be overlooked. The yields for biomass growth were 0.366 and 0.266 Cmol X/Cmol VFA for the fed-batch and batch assays, respectively. The suspended solids concentration also corroborates this, with increasing values from the beginning of the experiment to the moment when the maximum concentration of PHA was achieved. Values from 0.56 to 2.50 g VS/L and 0.87 to 1.19 g VS/L were achieved for the fed-batch and batch assays, respectively. Regarding the competition between growth and accumulation, the feeding regime also affected the amount of PHA stored and the HB:HV ratio, which in a further extent also affects the properties of the biopolymers.

### 3.4. Influence of the Feeding Strategy on the PHA Accumulation Stage

When VFAs are used as a substrate, it is common to perform an accumulation experiment feed in pulses to avoid the possible substrate inhibition by the substrate [42]. In these cases, the pulse concentration is fixed at conservative values (about 20 Cmmol/L). However, no information regarding the effects of the high VFAs concentrations is available, especially in the case of effluents containing also ammonia and NaCl.

In the present study, specific features associated to the use of fish-canning wastewater must be considered. When pulse wise feeding (pulses of 40 mL) is applied, then a progressive increase of the concentrations of NaCl and NH_4_^+^ occurs. However, when the feeding was supplied as a single pulse, the ammonium concentration progressively decreased due to biomass growth and the NaCl concentration remained constant throughout the experiment. In general terms, it can be said that the addition of the whole substrate at the beginning of the accumulation experiment (batch assay) provided better results than its addition in pulses (fed-batch assay) regarding the productivity but also the yield values. The most significant and relevant result is the PHA productivity, which is related to the economic feasibility of the process. The productivity was of 10.31 mg PHA/(L h) for the fed-batch assay while it was 55.4 mg PHA/(L h) for the batch assay (Table 4), which means that it was more than five times higher. Duque et al. (2014) obtained productivities between 150–560 mg PHA/(L h). These values are much higher than those obtained in the present study. This could be due to the inhibitory effect of the NaCl and the presence of large ammonium concentrations, which enhances the biomass growth. Having a look at Table 5, the obtained accumulation rates (0.006 Cmol PHA/(Cmol X h) and 0.071 Cmol PHA/(Cmol X h)) are quite low in comparison with other authors. They obtained values in the range of 0.15–0.47 Cmol PHA/(Cmol X h) for real fermented wastewaters such as cheese whey or sugar cane molasses, where no inhibitors or excess of nutrients were present [20,43]. With respect to the salinity, an IC50 of approximately 5 g NaCl/L for PHA accumulation has been reported [27] and the concentration in the present study was at maximum 8 g NaCl/L in both accumulation experiments, which supports the idea of having NaCl inhibition in both accumulations.

The yield of the process was also improved when operating in one single pulse mode. The batch experiment beat the fed-batch one as the yields were significantly higher for both monomers, with values of 0.029 Cmol HB/Cmol VFA and 0.005 Cmol HV/Cmol VFA for the fed-batch assays and 0.125 Cmol HB/Cmol VFA and 0.008 Cmol HV/Cmol VFA. Nonetheless, the most relevant change was observed for the HB yield while the HV yield remained nearly constant. This can be attributed to the presence of NaCl since its presence promotes the HB accumulation over the HV accumulation since propionate leads to higher energy release [27]. It is usual to find low yield values when fed-batch experiments are performed without nutrient limitation. Values of 0.08 Cmol HB/Cmol VFA [21] were obtained under nutrient excess using fermented paper mill wastewaters as a substrate.

As indicated previously, the operation of the accumulation assays was marked for the excess of NH_4_^+^ due to the use of fermented fish-canning wastewaters. Bengtsson et al. (2008) obtained better results for the nutrients limitation case, since 42.7 wt% of PHA was achieved under nutrient starvation conditions while 31.9 wt% of PHA were obtained under nutrient excess. Contradictory results were obtained by other authors: Johnson at al. (2010) observed that higher C/N ratios led to better accumulation performance while Moralejo et al. (2013) stated that the C/N ratio did not influence the maximum biopolymer accumulation once an appropriated enrichment is produced under nitrogen limiting conditions. The first authors used acetate as feeding supplied in pulses while Moralejo et al. (2013) used glycerol as a substrate fed in a one single pulse. None of the authors considered the influence of the feeding regime in their experiments. However, looking at the accumulation and growth yields obtained in the present study, it can be observed that the feeding strategy influences the accumulation performance. Growth is promoted in the fed-batch experiment since a higher yield value (0.366 Cmol X/Cmol VFA) and lower accumulation value (0.034 Cmol PHA/Cmol VFA) are obtained in comparison with the batch experiment (0.226 Cmol X/Cmol VFA and 0.133 Cmol PHA/Cmol VFA). This means that less substrate was directed to growth when a solely pulse with a high VFA concentration is added to the reactor and, subsequently, less competition between biomass growth and biopolymer accumulation would be expected. In any case, in order to avoid troubling competition between growth and accumulation, more experiments based in one single VFA pulse should be performed so as to establish the optimal accumulating conditions for the obtainment of biopolymers under the excess of nutrients.

Summing up, for inhibitory substrates such as fish-canning waste, it seems like it is better to feed in one single pulse than in pulse wise feeding since higher productivities and higher yields are achieved, as it has been previously discussed in this Section. However, further research is needed at this point to improve the results. For example, an enrichment operating under higher saline conditions is expected to provide better productivity values and to influence the composition of the obtained biopolymers. In general, it can be said that the enriched MMC is expected to be the key factor so as to improve the kinetic and stoichiometric parameters in the subsequent accumulation stage. Then, another key point is to study the influence of the cycle length in relationship with the C/N ratio. Finally, choosing between batch and fed-batch mode for the accumulation stage is another key factor too, as it will further improve the productivity of the process.

## 4. Future Perspectives

PHAs have been considered lately as promising candidates to substitute conventional plastics due to their biodegradability and to the possibilities of developing a full biobased production process. Particularly, microbial mixed cultures aim to reduce the production costs while trying to reach a high PHA productivity.

In pure cultures, halophiles are seen as one of the most promising strains for PHA production and make this biotech industry competitive. The main reason is that halophiles can be used in an unsterile process while accumulating relevant amounts of biopolymer and allowing for a clean PHA extraction process with water. In the case of mixed microbial communities, they would not be relevant on the unsterile process side, but they are of interest as the process downstream would be eased. The presence of NaCl is also known to affect the PHA properties as it increases the HB:HV ratio but also improves the crystallinity and the molecular weight [28]. It can be seen that halophile communities can help to develop a new way of producing sustainable bioplastics in both pure and mixed cultures.

However, more research is needed in this field. Likewise, more studies are needed for PHA production using rich-protein substrates. The influence of the operational conditions during the enrichment and the evolution of the microbiota are key factors to devise how to produce PHAs successfully under high ammonium concentrations. The accumulation batches also need to be optimised because parameters such as the feeding mode (batch or fed-batch) can influence the competition between growth and accumulation, as was seen in this study.

## 5. Conclusions

An MMC was enriched using a mixture of VFAs obtained from the acidification of fish-canning wastewater at 3.19 ± 0.79 g NaCl/L. Using this enriched biomass, two different PHA accumulations were performed: fed-batch and batch. The results from the accumulation assays show a PHA accumulation of 8.35 wt% PHA in the fed-batch and 5.70 wt% PHA in the batch. Nevertheless, when comparing the productivities, the obtained values were 55.4 mg PHA/(L h) for the batch assay and 10.31 mg PHA/(L h) in the fed-batch assay. This result is very relevant together with the fact that the biomass growth was more favoured in the fed-batch assay (0.366 Cmol X/Cmol VFA) rather than in batch assay (0.226 Cmol X/Cmol VFA). Nevertheless, the operational parameters must be optimized to improve the accumulation results as findings are promising despite the inhibitory conditions.

## Figures and Tables

**Table 1:** Fish-canning wastewater (FCW) characterization.

Parameter		Wastewater
Fats	g/L	1.2 ± 0.1
Proteins	g/L	7.3 ± 2.5
Carbohydrates	g/L	0.15 ± 0.03
VFA	g/L	11.3 ± 3.3
COD	g/L	28.4 ± 4.0
BOD_5_	g/L	17.9 ± 0.3
TSS	g/L	2.2 ± 0.9
VSS	g/L	1.6 ± 0.7
VSS/TSS	%	70.4 ± 6.8
NaCl	g/L	19.8 ± 5.9
**Ions**		
Na^+^	g/L	8.2 ± 2.8
K^+^	g/L	2.2 ± 0.8
Mg^2+^	g/L	n.d.
Ca^2+^	g/L	n.d.
SO_4_^2−^	g/L	0.9 ± 0.2
PO_4_^3−^	g/L	2.0 ± 0.9
Cl^−^	g/L	12.0 ± 3.5
NO_2_^−^	g/L	0.07 ± 0.02
NO_3_^−^	g/L	0.36 ± 0.04

n.d. = not detected.

**Table 2:** Average values of the operational parameters of the acidification reactor.

Parameter		CSTR
Operational conditions		
Operation days	d	203
OLR	g COD/(L · d)	12.3 ± 3.5
HRT	d	2
SRT	d	2
Operational parameters at steady state		
pH		7.5 ± 0.1
NH_4_^+^	g/L	4.4 ± 1.6
COD	g/L	28.3 ± 8.7
TSS	g/L	2.5 ± 0.7
VSS	g/L	1.5 ± 0.4
VSS/TSS	%	62.3 ± 6.9
NaCl	g/L	28.2 ± 10.1
VFA_TOTAL_	g COD/L	25.0 ± 4.6
HAc	g COD/L	10.9 ± 1.4
HPr	g COD/L	4.1 ± 1.0
HBu	g COD/L	8.1 ± 3.2
HVa	g COD/L	1.9 ± 0.5
TA	g CaCO_3_/L	11.5 ± 0.9
PA	g CaCO_3_/L	3.6 ± 0.4
Ripley ratio		2.3 ± 0.3
Salinity and other relevant ions		
Na^+^	g/L	10.9 ± 4.0
K^+^	g/L	1.4 ± 0.5
Mg^2+^	g/L	n.d.
Ca^2+^	g/L	n.d.
SO_4_^2^^−^	mg/L	542.4 ± 153.6
PO_4_^3^^−^	mg/L	636.3 ± 245.4
Cl^−^	g/L	13.8 ± 2.8
NO_2_^−^	mg/L	49.1 ± 16.8
NO_3_^−^	mg/L	304.1 ± 67.4

n.d. = not detected.

**Table 3:** Average values of the operational parameters of the enrichment reactor.

Parameter		Enrichment SBR
Operational conditions		
Operation time	d	384
HRT	d	1
SRT	d	1
Cycle	h	12
Operational parameters in steady state at the end of the feast phase		
DO_SATURATION_	mg/L	7.2 ± 0.1
T	°C	30.9 ± 1.0
pH		8.7 ± 0.4
NH_4_^+^	mg/L	256.4 ± 20.3
TS	g/L	1.34 ± 0.47
VS	g/L	1.21 ± 0.43
VS/TS	%	90.3 ± 5.2

**Table 4:** Summary of the results obtained from the accumulation assays.

Experiment	HB	HV	PHA	HB:HV	Productivity	TS	VS
	wt%	wt%	wt%	g/g	mg PHA/L h	g/L	g/L
Fed-batch (0 h)	2.21	2.33	4.54	0.95	-	0.72	0.56
Fed-batch (27 h)	7.14	1.21	8.35	5.91	10.31	3.29	2.50
Batch (0 h)	0.12	0.01	0.13	15.3	-	1.71	0.87
Batch (2.7 h)	5.36	0.34	5.70	15.9	55.4	2.59	1.19
Batch (24 h)	0.09	0.00	0.09	-	-	3.66	2.12

**Table 5:** Kinetic and stoichiometric parameters obtained from the accumulation assays.

	−q_VFA_	q_PHA_	q_X_	q_CO2_	Y_PHB_	Y_PHV_	Y_PHA_
	Cmol/ Cmol X h	Cmol/ Cmol X h	Cmol/ Cmol X h	Cmol/ Cmol X h	Cmol/ Cmol VFA	Cmol/ Cmol VFA	Cmol/ Cmol VFA
Fed-batch	0.187	0.006	0.068	0.064	0.034	0.366	0.345
Batch	0.536	0.071	0.121	0.372	0.133	0.226	0.694

## Data Availability

The data presented in this study are available on request from the corresponding author.
